# Supplementary material for: One-shot entorhinal maps enable flexible navigation in novel environments
Source: Nature. 2024 Oct 9;635(8040):943–50. doi: 10.1038/s41586-024-08034-3 (PMC11602719; doi:10.1038/s41586-024-08034-3)
Supplement: Supplementary file 1 — A detailed tabulation of all the animals included in the study and the sessions run by each. [file 41586_2024_8034_MOESM1_ESM.pdf]

nature portfolio

https://doi.org/10.1038/s41586-024-08034-3

Supplementary information

One-shot entorhinal maps enable flexible navigation in novel environments

In the format provided by the authors and unedited

Animal	# Build-Up Track	# of Random Environment	# of Hidden Reward	# of Hidden Reward with Muscimol and Neuropixels Recording	# of Hidden Reward with Muscimol and no Neuropixels Recording	# of Hidden Reward with Fluorescent Muscimol and Neuropixels Recording	# of Hidden Reward with Fluorescent Muscimol and no Neuropixels Recording	# of Hidden Reward with Saline and Neuropixels Recording	# of Hidden Reward with Saline and no Neuropixels Recording	# of Fluorescent Muscimol only	# of Open Field and 1D Dark
M2	6	0	0	0	0	0	0	0	0	0	0
N1	5	0	0	0	0	0	0	0	0	0	0
N2	5	0	0	0	0	0	0	0	0	0	0
P3	3	0	0	0	0	0	0	0	0	0	0
AF3	0	3	0	0	0	0	0	0	0	0	0
AF4	0	4	0	0	0	0	0	0	0	0	0
AG1	0	3	6	0	0	0	0	0	0	0	0
AJ2	0	3	0	1	1	0	0	1	2	0	0
AK1	0	3	1	0	0	0	1	1	0	0	0
AK2	0	2	1	0	0	1	0	1	0	0	0
AL1	0	2	0	0	0	0	0	0	0	0	0
AM1	0	4	3	2	0	0	0	2	0	0	0
AM2	0	6	2	1	0	0	0	2	0	0	0
AN1	0	3	1	1	0	0	0	2	0	0	0
AN2	0	5	2	2	0	0	0	2	0	0	0
musf1	0	0	0	0	0	0	0	0	0	1	0
musf2	0	0	0	0	0	0	0	0	0	1	0
musf5	0	0	0	0	0	0	0	0	0	1	0
musf6	0	0	0	0	0	0	0	0	0	1	0
AO1	0	0	0	0	0	0	0	0	0	0	1
AO2	0	0	0	0	0	0	0	0	0	0	1
AO5	0	0	0	0	0	0	0	0	0	0	1
	# of build-up track sessions	# of random environment sessions	# of hidden reward sessions	# of hidden reward sessions with muscimol and neuropixels	# of hidden reward sessions with muscimol and no neuropixels	# of hidden reward sessions with fluorescent muscimol and neuropixels	# of hidden reward sessions with fluorescent muscimol and no neuropixels	# of hidden reward sessions with saline and neuropixels	# of hidden reward sessions with saline and no neuropixels	# of fluorescent muscimol only sessions	# of open field and 1D dark sessions
	19	38	16	7	1	1	1	11	2	4	3
	# of animals build-up track	# of animals random environment	# of animals hidden reward	# of animals hidden reward with muscimol and neuropixels	# of animals hidden reward with muscimol and no neuropixels	# of animals hidden reward with fluorescent muscimol and neuropixels	# of animals hidden reward with fluorescent muscimol and no neuropixels	# of animals hidden reward with saline and neuropixels	# of animals hidden reward with saline and no neuropixels	# animals with fluorescent muscimol	# of animals open field and 1D dark
	4	11	7	5	1	1	1	7	1	6	3

Supplementary Table 1: Tabulation of all the animals and sessions they ran in this manuscript. The top half of the table enumerates the number of sessions a given animal contributed to each experiment. The bottom half of the table summarizes the total number of animals and sessions for each experiment.
