# Supplementary material for: One-shot entorhinal maps enable flexible navigation in novel environments
Source: Nature. 2024 Oct 9;635(8040):943–50. doi: 10.1038/s41586-024-08034-3 (PMC11602719; doi:10.1038/s41586-024-08034-3)
Supplement: Supplementary file 2 — Reporting Summary [file 41586_2024_8034_MOESM2_ESM.pdf]

nature portfolio

Corresponding author(s): Lisa Giocomo

Last updated by author(s): Sep 30, 2024

Reporting Summary

Nature Portfolio wishes to improve the reproducibility of the work that we publish. This form provides structure for consistency and transparency in reporting. For further information on Nature Portfolio policies, see our Editorial Policies and the Editorial Policy Checklist.

Statistics

For all statistical analyses, confirm that the following items are present in the figure legend, table legend, main text, or Methods section.

n/a	Confirmed	
☐	☒	The exact sample size (n) for each experimental group/condition, given as a discrete number and unit of measurement
☐	☒	A statement on whether measurements were taken from distinct samples or whether the same sample was measured repeatedly
☐	☒	The statistical test(s) used AND whether they are one- or two-sided<br>*Only common tests should be described solely by name; describe more complex techniques in the Methods section.*
☐	☒	A description of all covariates tested
☐	☒	A description of any assumptions or corrections, such as tests of normality and adjustment for multiple comparisons
☐	☒	A full description of the statistical parameters including central tendency (e.g. means) or other basic estimates (e.g. regression coefficient) AND variation (e.g. standard deviation) or associated estimates of uncertainty (e.g. confidence intervals)
☐	☒	For null hypothesis testing, the test statistic (e.g. F, t, r) with confidence intervals, effect sizes, degrees of freedom and P value noted<br>*Give P values as exact values whenever suitable.*
☒	☐	For Bayesian analysis, information on the choice of priors and Markov chain Monte Carlo settings
☒	☐	For hierarchical and complex designs, identification of the appropriate level for tests and full reporting of outcomes
☐	☒	Estimates of effect sizes (e.g. Cohen's d, Pearson's r), indicating how they were calculated

Our web collection on statistics for biologists contains articles on many of the points above.

Software and code

Policy information about availability of computer code

Data collection	Custom code was written for Unity 5.6.3p2 for virtual reality experiments. Neuropixels data were collected using spikeGLX.
Data analysis	All custom code is available at the following website: https://github.com/GiocomoLab/mec-rapid-learning/tree/main.<br>Kilosort2 was used for spike sorting offline.<br>Phy version 1 was used to manually inspect and curate clusters after spike sorting.<br>SHARP-Track was used to reference histological slices to the Allen Brain Atlas and to infer probe locations from dyes.<br>DeepLabCut version 2.2.0.6 was used to identify animals' position and head direction from green and red LEDs on a headstage holder.

For manuscripts utilizing custom algorithms or software that are central to the research but not yet described in published literature, software must be made available to editors and reviewers. We strongly encourage code deposition in a community repository (e.g. GitHub). See the Nature Portfolio guidelines for submitting code & software for further information.

Data

Policy information about availability of data

All manuscripts must include a data availability statement. This statement should provide the following information, where applicable:

- Accession codes, unique identifiers, or web links for publicly available datasets
- A description of any restrictions on data availability
- For clinical datasets or third party data, please ensure that the statement adheres to our policy

All data required to reproduce the paper figures are available in two parts on Mendeley Data:

Wen, John; Sorscher, Ben; Giocomo, Lisa (2024), "One-shot entorhinal maps enable flexible navigation in novel environments - part 1", Mendeley Data, V1, doi: 10.17632/rgtk6jygjc.1

Wen, John; Sorscher, Ben; Giocomo, Lisa (2024), "One-shot entorhinal maps enable flexible navigation in novel environments - part 2", Mendeley Data, V1, doi: 10.17632/2n4t9bw3xz.1

A publicly available database used to register histological sections to a reference mouse brain atlas is available here: https://figshare.com/articles/dataset/Modified_Allen_CCF_2017_for_cortex-lab_allenCCF/25365829

Research involving human participants, their data, or biological material

Policy information about studies with human participants or human data. See also policy information about sex, gender (identity/presentation), and sexual orientation and race, ethnicity and racism.

Reporting on sex and gender	No human data was used for this study.
Reporting on race, ethnicity, or other socially relevant groupings	No human data was used for this study.
Population characteristics	No human data was used for this study.
Recruitment	No human data was used for this study.
Ethics oversight	No human data was used for this study.

Note that full information on the approval of the study protocol must also be provided in the manuscript.

Field-specific reporting

Please select the one below that is the best fit for your research. If you are not sure, read the appropriate sections before making your selection.

☒ Life sciences ☐ Behavioural & social sciences ☐ Ecological, evolutionary & environmental sciences

For a reference copy of the document with all sections, see nature.com/documents/nr-reporting-summary-flat.pdf

Life sciences study design

All studies must disclose on these points even when the disclosure is negative.

Sample size	Sample sizes were determined in part by what is conventionally required in studies with mice. For each experiment, at least 4 different mice were used to ensure replication.
Data exclusions	Some analyses required a sufficient number of co-recorded grid cells from a module. These analyses are explicitly stated in the manuscript. Recording sessions that fail to meet this criterion were not used for these analyses.
Replication	Replication was verified by conducting the same experiment in different animals and across different days.<br>19 sessions were conducted across 4 animals on the build-up track.<br>38 sessions were conducted across 11 animals on the random environment track.<br>16 sessions were conducted across 7 animals on the hidden reward track.<br>3 sessions were conducted across 3 animals on the open field and 1D head-fixed dark runs.<br>10 muscimol sessions and 13 saline control sessions were conducted across 7 animals on the hidden reward track. Each of the seven animals injected with muscimol underwent at least one control saline session.<br>For more details, see the associated supplementary table.
Randomization	Generally, randomization of subjects into different groups was not relevant in our study as most comparisons were made within animal, and all animals used within an experiment were subject to the same behavioral and electrophysiological data collection protocol. However, for our

	inactivation studies using muscimol and saline control, which substance was used on the first day of recording was randomized across subjects.
Blinding	For most experiments, blinding was not necessary as there were no "treatment" groups. The only treatment compared muscimol to saline injections. The data from those experiments were analyzed blind, but because of the drastic effects of muscimol, far fewer recorded neurons were present in the muscimol-injected cases.

Reporting for specific materials, systems and methods

We require information from authors about some types of materials, experimental systems and methods used in many studies. Here, indicate whether each material, system or method listed is relevant to your study. If you are not sure if a list item applies to your research, read the appropriate section before selecting a response.

Materials & experimental systems

n/a	Involved in the study	
☒	☐	Antibodies
☒	☐	Eukaryotic cell lines
☒	☐	Palaeontology and archaeology
☐	☒	Animals and other organisms
☒	☐	Clinical data
☒	☐	Dual use research of concern
☒	☐	Plants

Methods

n/a	Involved in the study	
☒	☐	ChIP-seq
☒	☐	Flow cytometry
☒	☐	MRI-based neuroimaging

Animals and other research organisms

Policy information about studies involving animals; ARRIVE guidelines recommended for reporting animal research, and Sex and Gender in Research

Laboratory animals	Mice: C57Bl/6, between 12-24 weeks of age.
Wild animals	This study did not involve wild animals.
Reporting on sex	Only females were used for the virtual reality experiments in this study. This was for the following reasons: 1. In our lab, we have found that female mice typically learn faster than male counterparts in on our head-fixed virtual reality setup. 2. Female mice are less aggressive, reducing the chance of headbar detachment when on the head-fixed virtual reality setup. Three male mice were added during the revision stage to record the activity of freely moving mice. Male mice were used as they are larger and can better support the weight of the chronic implant.
Field-collected samples	This study did not involve samples collected from the field.
Ethics oversight	All procedures were approved by the Institutional Animal Care and Use Committee at Stanford University School of Medicine

Note that full information on the approval of the study protocol must also be provided in the manuscript.
